# Therapeutics of Bone Diseases*Read before International Hahnemannian Association.

**Published:** 1885-01

**Authors:** L. B. Wells

**Affiliations:** Utica, New York


### THERAPEUTICS OF BONE DISEASES.*

L. B. Wells, M. D., Utica, New York.

Bones, in their process of formation, although not endowed with the same degree of vitality of other organs of the body, are subject to the same laws of waste and supply peculiar to their own organization, as other parts of the animal organism.

Hence, when diseased, they are subject to structural changes peculiar to themselves. Being subject to established laws in their formation, they are not less so in a morbid condition.

We may therefore be assured that morbid changes will be made subservient to a correct application of therapeutic agencies.

*Aconite.*—Inflammation of the bones and periosteum, with swelling and dull aching. Restlessness.

*Agaricus.*—Pains in the long bones, as if bruised, after motion. Pains in left tibia. Pains in the spine between the vertebræ.

*Agnus castus.*—Inflammatory swelling of the joints. Gouty nodosities.

*Angustura.*—Caries, or very painful ulcers, deep seated in the bony structure.

*Apis mel.*—Periosteum inflamed.

*Asafœtida.*—Inflammation and caries of the bones involving the soft parts with ulcers with hardened edges. Softening of the bones with easy bleeding. Caries after the abuse of Mercury.

---

*Read before International Hahnemannian Association.

*Aurum.*—Secondary syphilis after abuse of Mercury. Looseness of the teeth, and ulcers of the gums and fetid breath. Caries of the palate and nasal bones. Bone pains at night, so severe that they cannot be borne.

*Argent. met.*—Acts on the cartilages and joints. Arthritic bruised pains in the joints.

*Arnica.*—Aching in the bones and periosteum.

*Baryta carb.*—Tearing and tension in the long bones. Boring in the bones.

*Berberis.*—Sensation of scraping at the bones. Cold sensation in the bones.

*Belladonna.*—Red shining swelling of the joints. Pains along the periosteum.

*Benzoic acid.*—Swelling of the knee-joints. Cracking in the knee-joint.

*Boletus laricis.*—Aching distress in all the joints.

*Bothrops lanceolatus.*—Caries of the bones.

*Cadmium sulph.*—Cutting pains of the joints. Caries of the bones of the nose.

*Calcarea carb.*—Curvature of the spine and long bones. Swelling and softening of the bones with curvature. Exostosis and caries of the bones of the extremities. Rachitis.

*Calcarea phos.*—Pains along the sutures and symphyses. Non-union of fractured bones. Curvature of the spine. Swelling of the condyles and arms and spina bifida. Rachitis. Open fontanelles.

*Camphora.*—Cracking of the joints.

*Capsicum.*—Joints crack; are stiff and painful.

*Carduus benedictus.*—Aching of all the bones after stretching the limbs.

*Cepa.*—Aching of the joints.

*Cinchona.*—Caries with profuse sweat.

*Cocculus.*—Gouty pains and cracking of the joints.

*Colchicum.*—Acts on the periosteum. Painful flexion of the joints.

*Cuprum met.*—Pains in the bones as if they would break.

*Curare.*—Periostitis.

*Cyclamen.*—Tearing pains in parts where the bones are near the surface.

*Digitalis.*—Piercing pains in the joints.

*Dulcamara.*—Exostosis. Scrofula.

*Ferrum.*—Cracking in the joints. Bones disposed to soften, or bend. Fractures unite slowly.

*Fluoric acid.*—Diseases of the long bones.

*Guarea.*—Stiffness of the trunk. Constriction of the back. Cutting pain in the sacrum. Caries of the bones. Swelling of the affected parts. Cracking in the joints. Nocturnal pain in the bones. Cutting pain in the joints. Bruised pain in the bones. Pain in the periosteum of the arm bones.

*Guiaicum.*—Rheumatic swelling of the joints. Aching of the bones with swelling. Syphilis. Caries and spongy affection of the bones. Pressing pains in the bones.

*Helleborus.*—Stinging boring in the periosteum in cool air.

*Hepar sulph.*—Caries. Hard burning nedosities.

*Iodium.*—Nightly bone pains. Arthritic affection of the joints.

*Kali bich.*—Scrofula. Secondary syphilis, with diseases of the bones. Necrosis. Exostosis. Pains worse at night.

*Kali carb.*—Caries. Bones feel bruised. Cracking in the joints on motion. Rheumatic pains in the joints.

*Kali iodatum.*—Diseases of the periosteum and capsular ligaments of the joints.

*Kalmia latifolia.*—Acute rheumatism from joint to joint, frequently changing.

*Kreosotum.*—Rheumatic pains in the joints.

*Lactic acid.*—Rheumatic pains in the bones, worse on motion.

*Lithium carb.*—Arthritis. Bones, joints, and muscles sore as if beaten.

*Lycopodium.*—Bones inflamed, mostly the ends with nocturnal pains. Softening of the bones.

*Manganum aceticum.*—Inflammation of the bones with nightly insupportable digging pains. Inflammation of the joints with digging pains at night.

*Mercurius.*—Bone diseases, worse at night.

*Mercurius corros. sub.*—Necrosis of upper jaw. Inflammation of periosteum.

*Mezereum.*—Bones inflamed, swollen, especially shafts of cylindrical bones. Caries after the abuse of Mercury. Bones feel distended.

*Natrum sulph.*—Cracking of the joints, knees stiff. Pain in the bones. Sycosis.

*Nitric acid.*—Caries. Cracking in the joints.

*Nitri spi. dul.*—Striking boring in the bones of the face, back, and various parts of the body, tips of the toes, knees, and cranial bones soon after taking the dose. Drawing in the cranial bones, ankles, and toes.

*Phosphorus.*—Swelling of the bones. Necrosis, especially lower jaw. Exostosis, especially of the skull. Tearing boring pains. Disease of the hip joint.

*Phosphoric acid.*—Interstitial inflammation of the bones, scrofulus, syphilitic, or mercurial. Caries with smarting pains. Inflammation of the periosteum with gnawing and burning pains. Pains in the bones at night.

*Phytolacca.*—Bones inflamed, swollen ; joints red and swollen. Periosteum affected in mecurialism and syphilis.

*Psorinum.*—Caries.

*Pulsatilla.*—Jerking tearing in the bowels. Scraping or tingling in the periosteum.

*Rhus tox.*—Inflammation and swelling of the long bones. Pains, as if the flesh were torn loose from the bones, or as if the bones were being scraped.

*Raphanus.*—Pain in bones when touched, the bones of the left orbit, the nasal and maxillary bones on the left side. Numbness of the part near the painful bones. Pain in the vertebral column as if a foreign body passed through it from top to bottom.

*Ruta grav.*—Bruises and other mechanical injuries of the periosteum and bones. Periostitis.

*Sabadilla.*—Boring cutting in the bones. Intense pains in all the bones, especially in the joints, as if the interior of the bones were cut or scraped with a knife.

*Sabina.*—Drawing pains through the long bones. Tearing and stinging in the joints after they become swollen, worse in the heated room, better in cool air or cool room.

*Sarsaparilla.*—Scrofulous diseases in general.

*Silicea.*—Inflammation, swelling, ulceration and necrosis of bones.

*Solanum.*—Pains in all the bones.

*Strontiana-carb.*—Pains in the long bones and in the marrow.

*Staphisagria.*—Swelling and suppuration of the bones, also of the periosteum. Arthritic nodosities of the joints.

*Sulphur.*—Scrofulous and rickety complaints.

*Theridion.*—Scrofula, when other remedies fail. Rachitis, caries, necrosis. Bones pain as if they would fall asunder.

*Thuja.*—Flesh feels as if beaten off the bones.

*Triosteum.*—Aching in all the bones. Stiffness of all the joints of the upper as well as the lower extremities.

*Vinca.*—Arthritic tearing in the bones.

---

The Medical Advance.—With its January issue the *Advance* will commence the publication of a thirty-two paged addition, devoted to diseases of women and children, etc., under the able editorship of Dr. Phil. Porter.

